# Long-Chain Hydrocarbons in the Mucous Layer of the *Galleria mellonella* Insect Eggs as Potential Antibacterial Agents against Multidrug-Resistant Bacteria

**DOI:** 10.1021/acsomega.5c00938

**Published:** 2025-04-28

**Authors:** Letícia F. Luz, Gabriela L. Nascimento, Gabrielle N. Volcan, Rosane A. Ligabue, Gabriela M. Miranda, Danielle S. Trentin

**Affiliations:** 1 Graduate Program in Biosciences, Federal University of Health Sciences of Porto Alegre, Sarmento Leite, 245, Porto Alegre, 90050-170 Rio Grande do Sul, Brazil; 2 Undergraduate Nursing Course, Federal University of Health Sciences of Porto Alegre, Sarmento Leite, 245, Porto Alegre, 90050-170 Rio Grande do Sul, Brazil; 3 Undergraduate Pharmacy Course, Federal University of Health Sciences of Porto Alegre, Sarmento Leite, 245, Porto Alegre, 90050-170 Rio Grande do Sul, Brazil; 4 Graduate Program in Materials Engineering and Technology, Pontifical Catholic University of Rio Grande do Sul, Ipiranga, 6681, Porto Alegre, 90619-900 Rio Grande do Sul, Brazil

## Abstract

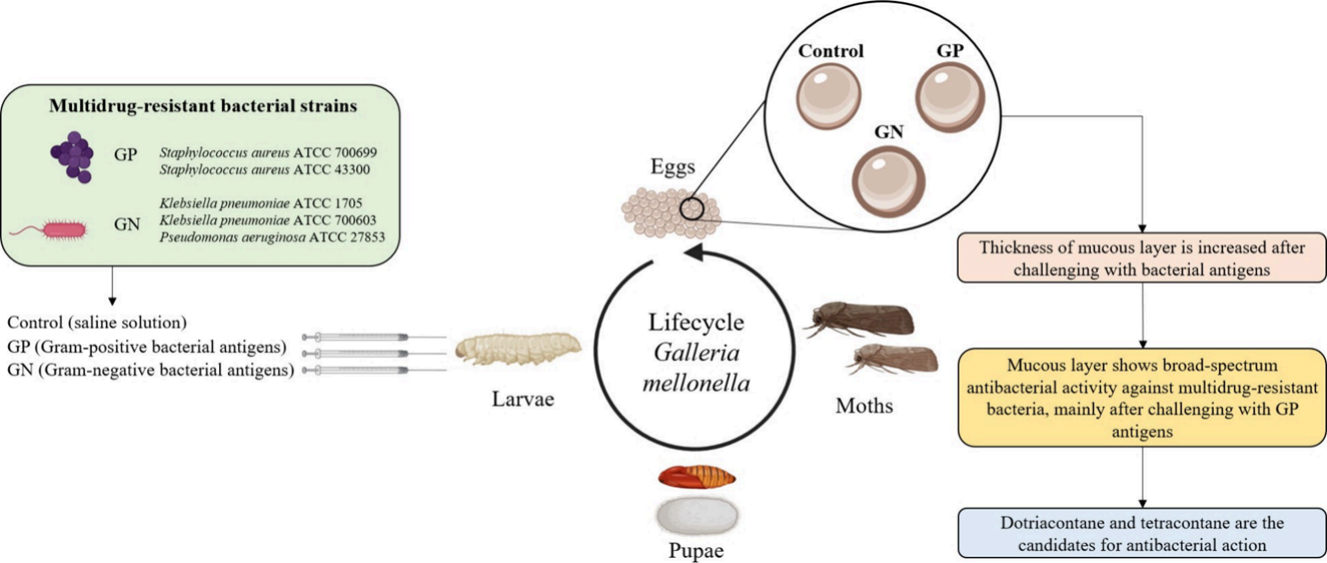

Natural products represent a vital source of chemical entities for the development of anti-infective agents. Insects face constant threats from pathogens and have evolved diverse mechanisms of the infection response. Among various insect species, the chemical protection provided by *Galleria mellonella* eggs against microorganisms remains poorly understood. This study aimed to investigate whether *G. mellonella* produces chemical compounds that could serve as anti-infective agents against clinically important bacteria. Additionally, the study examined the effects of larval exposure to bacterial antigens from multidrug-resistant *Staphylococcus aureus*, *Klebsiella pneumoniae*, and *Pseudomonas aeruginosa*on the chemical composition, morphology, and anti-infective properties of the eggs. Larvae were challenged with antigens derived from multidrug-resistant Gram-positive and Gram-negative bacteria. Eggs from intragroup mating were collected and analyzed by using histological and physicochemical techniques, including field-emission gun scanning electron microscopy, energy-dispersive X-ray spectroscopy, and Fourier-transform infrared spectroscopy. Antibacterial and antibiofilm activities of the egg extracts were assessed using broth microdilution and crystal violet assays, respectively. The volatile compound profile of the extracts was characterized by gas chromatography–mass spectrometry. This pioneering study demonstrates the broad-spectrum antibacterial activity of *G. mellonella* eggs against clinically relevant bacteria. Notably, the antibacterial efficacy of the mucous layer extract was significantly enhanced when larvae were exposed to Gram-positive bacterial antigens. Dotriacontane and tetracontane were identified as the predominant volatile compounds. These findings highlight *G. mellonella* eggs as a promising source of bioactive compounds and underscore the potential of long-chain hydrocarbons in the development of novel antibacterial agents.

## 1 Introduction

Insects constitute the largest and most diverse group of organisms on Earth, accounting for 80–90% of global biodiversity. They employ various chemical tools for survival, including the production of numerous substances to defend against pathogen attacks. However, only a small proportion of insect species have been chemically analyzed or explored for the presence of potentially medicinally relevant substances.^1^ In this context, research on antimicrobial peptides led to the development of OMN6, a cyclic peptide derived from cecropin (an insect peptide). OMN6 binds to and penetrates bacterial membranes, resulting in bacterial death.^2^ Currently, OMN6 is undergoing clinical evaluation for the treatment of infections caused by carbapenem-resistant *Acinetobacter baumannii*,^3^ highlighting recent advancements in the field and its potential contribution to future antimicrobial therapies.

Antimicrobial resistance is a global concern, ranked among the top 10 challenges facing humanity. Projections indicate that, by 2050, antimicrobial resistance could result in more than 10 million deaths annually if current trends persist.^4^ Moreover, infections caused by multidrug-resistant bacteria are associated with higher mortality rates and financial costs compared to those caused by antibiotic-sensitive bacteria.^3^*Pseudomonas aeruginosa*, *Klebsiella pneumoniae*, and multidrug-resistant *Staphylococcus aureus* are listed among the priority pathogens for research and development of new antibacterial agents.^5^ Current antibacterial research follows two primary approaches: (i) traditional strategies focused on molecules that inhibit bacterial viability and (ii) nontraditional strategies aimed at mitigating bacterial virulence, such as biofilm formation and toxin production, without inhibiting bacterial growth.^3^ Biofilm refers to a microbial lifestyle in which free-floating microorganisms adhere to a surface and initiate the formation of sessile microcolonies surrounded by a self-produced extracellular matrix, functioning as a microbial community. This phenotype provides microorganisms with protection and resistance against various threats, including extreme environments, ultraviolet radiation, extreme pH, high temperatures, high salinity, high pressure, malnutrition, immune system responses, and antibiotics. Biofilm-associated bacteria have been implicated in more than 80% of all human infections, particularly chronic infections and those related to medical devices.^6,7^

The literature reports antibacterial activity in insect eggs from certain species, such as (i) the serosa layer of *Tribolium castaneum* (Coleoptera: Tenebrionidae), effective against *Escherichia coli*and *Micrococcus luteus*;^8^ (ii) antimicrobial peptides from *Tenebrio molitor* (Coleoptera: Tenebrionidae), active against Gram-positive bacteria such as *Arthrobacter globiformis*, *Bacillus subtilis*, and *Bacillus thuringiensis*;^9^ and (iii) chitosan from *Bombyx mori* (Lepidoptera: Bombycidae), effective against *S. aureus*, *Bacillus cereus*, *E. coli*, and *K. pneumoniae*.^10^ However, the protective mechanisms of insect eggs against pathogens remain largely unknown for most species, including *Galleria mellonella*.

The insect *Galleria mellonella* (Lepidoptera: Pyralidae) undergoes four developmental stages: egg, larva, pupa, and moth.^11−14^ Its eggs exhibit small variations in size (approximately 478 μm in length and 394 μm in width), shape (spheroidal, ellipsoid, or ovoid), and color (ranging from pinkish to creamy white to white). The surface of the eggs is externally characterized by hexagonal and pentagonal patterns with a rough texture.^13−15^ A mucous substance, produced by female accessory glands, covers the eggs, serving to protect them from pathogens.^15−17^ Beneath this mucous layer lies the chorion, which predominantly consists of cysteine-rich proteins, followed by the vitelline envelope and a granular yolk.^18^

The immune system of insects consists of innate responses comprising two lines of defense against pathogens: (i) the cuticle, a complex physicochemical barrier that protects them from the external environment and (ii) the hemolymph, analogous to mammalian blood, which is involved in the transport of nutrients, waste products, and signaling molecules and contains both cellular and humoral defense mechanisms that respond to a variety of microorganisms. The cellular response is mediated by hemocytes with phagocytic activity, eliminating pathogens through phagocytosis, while the humoral response involves lytic enzymes, antimicrobial peptides, opsonins, and melanin, which act synergistically to destroy invading microorganisms.^19,20^ Insects lack an immune system equivalent to the adaptive antibody-mediated responses observed in vertebrates. However, some species have evolved a process known as immune priming, which occurs when a dose of killed microorganisms or a sublethal dose of live pathogens is introduced into the host. This process activates cellular and humoral immune responses, offering protection against subsequent challenges and enabling resistance to infections that would otherwise be fatal.^21−23^

Therefore, this study aimed to investigate whether *G. mellonella* produces chemical compounds that could serve as anti-infective agents against clinically important bacteria. Additionally, it evaluated the effects of exposing larvae to bacterial antigens from multidrug-resistant *Staphylococcus aureus*, *Klebsiella pneumoniae*, and *Pseudomonas aeruginosa*on the chemical composition, morphology, and anti-infective properties of the eggs.

## 2 Results and Discussion

### 2.1 Morphological and Physicochemical Characterization of *G. mellonella* Eggs

**Figure 1:**
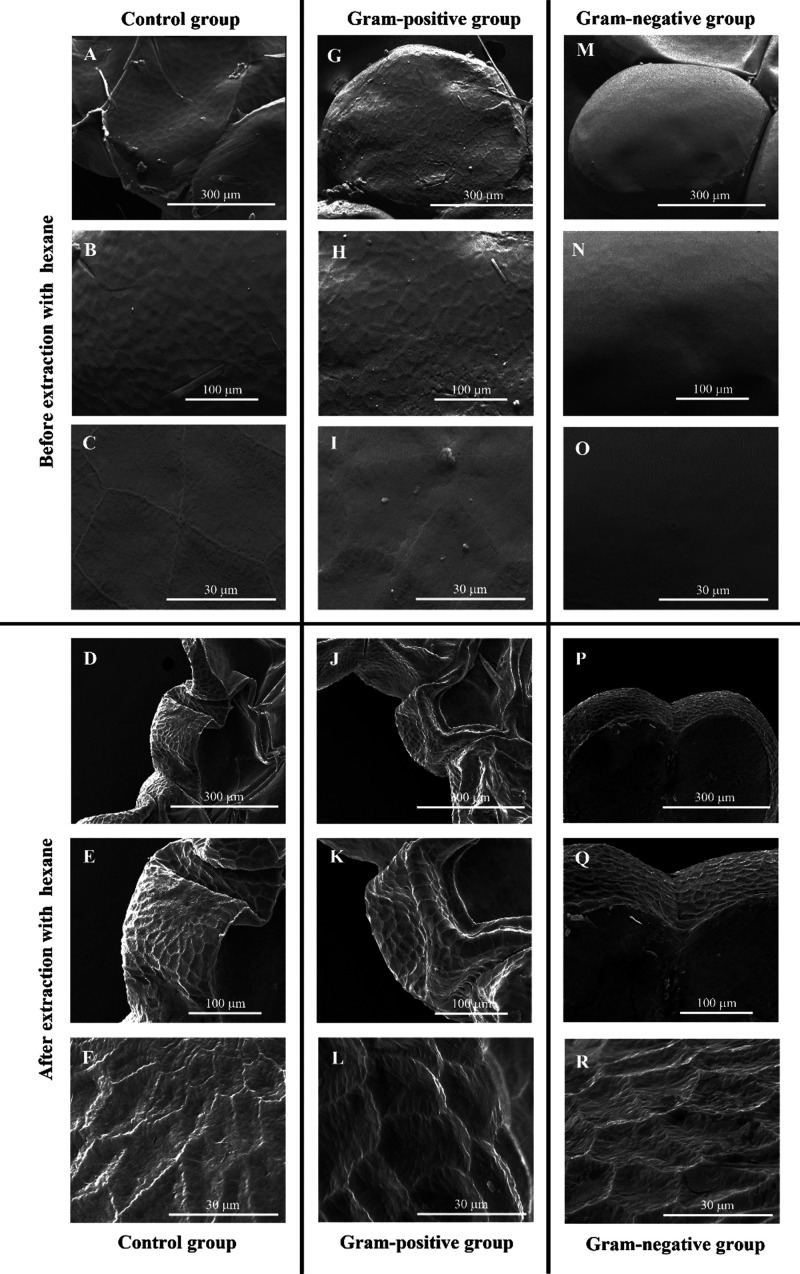
FEG-SEM images of the *G. mellonella* eggs surface before and after extraction with hexane: eggs from the control group before (A–C) and after (D–F) hexane extraction; eggs from larvae exposed to Gram-positive antigens before (G–I) and after (J–L) hexane extraction; and eggs from larvae exposed to Gram-negative antigens before (M–O) and after (P–R) hexane extraction. Scale bars in images (A, D, G, J, M, P): 300 μm; (B, E, H, K, N, Q): 100 μm; (C, F, I, L, O, R): 30 μm.

The FEG-SEM analysis showed that the surface of the control group eggs presented a classical hexagonal and pentagonal design and a rough texture due to the thin layer (Figure 1A–C). The literature points out that *G. mellonella* eggs are coated by a mucous layer, which is produced in the female moth’s colleteric glands during oviposition and acts as a protective barrier against pathogens.^15−17^ Changes in the mucous layer occurred in the eggs from larvae exposed to Gram-positive (Figure 1G–I) and Gram-negative antigens (Figure 1M–O) when compared to control eggs (Figure 1A–C). These changes were evidenced by a smooth and free from hexagonal and pentagonal geometry surface, especially in the eggs from the Gram-negative group (Figure 1M–O). A preliminary evaluation of the response of *G. mellonella* eggs (control and exposed to bacterial antigens) to *S. aureus* demonstrated that most of their surface was not covered by bacterial cells, indicating that the mucous layer of the eggs protects them against microbial attachment (Figure S1), supporting our research hypothesis.

The surfaces of the eggs obtained from all groups of animals were also evaluated after the hexane extraction process. The texture of the residual eggs from all groups showed greater evidence of hexagonal and pentagonal geometry, clearer shape, and drier appearance than eggs before extraction (Figure 1D–F,J–L,P–R). Moreover, eggs from the challenged groups (Figure 1J,K,P–R) showed the same surface topography as the control group (Figure 1D–F), indicating that the mucous layer was completely removed.

**Figure 2:**
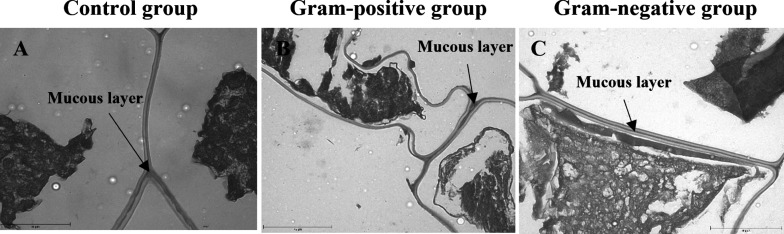
Histological images of *G. mellonella* eggs (A–C). Arrows point to the mucous layer of the eggs from the control group (A), of the eggs from animals challenged with Gram-positive bacterial antigens (B), and of the eggs from animals challenged with Gram-negative bacterial antigens (C). Scale bars in images: 75 μm.

The difference visualized in the egg’s surface between control and challenged groups, before hexane extraction, was reinforced by histological analysis (Figure 2). There was an increase in the thickness of the mucous layer from 0.82 ± 0.12 μm in the control group (Figure 2A) to 1.34 ± 0.28 and 1.97 ± 0.41 μm for eggs from animals challenged with Gram-positive and Gram-negative antigens, respectively (Figure 2B,C).

Furthermore, EDS analysis showed an increase in nitrogen content from 9.4% in the control group to 15.4 and 17.3% for eggs from animals challenged with Gram-positive and Gram-negative bacterial antigens, respectively (Figure S2 and Table 1).

**Table 1:** Percentage of Chemical Elements Present on the Surface of *G. mellonella* Eggs Obtained from Control Animals and Challenged with Bacterial Antigens (Gram-Positive and Gram-Negative) through EDS Evaluation^a^

	**weight (%)**		
**element**	**control group**	**Gram-positive group**	**Gram-negative group**
C	62.8	48.8	49.4
N	9.4	15.4	17.3
O	25.6	27.9	28.3

a C: carbon; N: nitrogen; O: oxygen.

**Figure 3:**
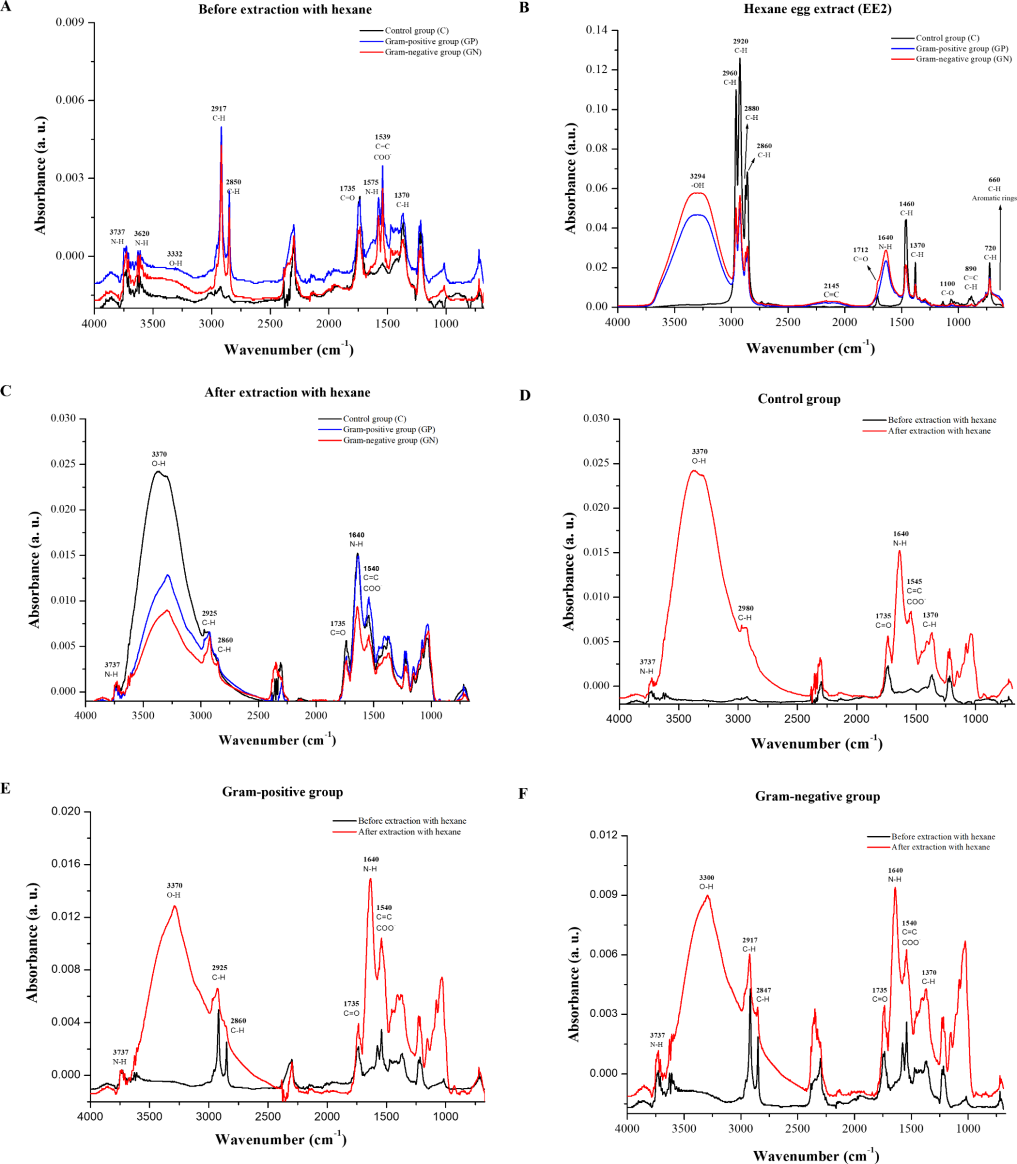
FTIR spectra of the *G. mellonella* eggs. Eggs obtained from the control group larvae (black line), Gram-positive group (blue line), and Gram-negative group (red line) before (A) and after (C) the extraction process with hexane. Panel (B) refers to the FTIR spectra of hexane egg extract (EE2) obtained from all groups. Panels (D–F) refer to the comparison between FTIR spectra before (black lines) and after (red lines) hexane extraction: control group (D), Gram-positive group (E), and Gram-negative group (F).

Since this is a pioneer study on *G. mellonella* eggs’ chemical composition, all the main bands obtained by FTIR analysis were assigned based on the infrared spectroscopy literature.^24−27^ The structural characteristics of the eggs are remarkably distinct in the FTIR spectra when the eggs from the control group are compared with eggs from groups challenged with bacterial antigens (Figure 3A). The intensity of the bands at the 3750–3600 region and at 3332 cm^–1^ (N–H and O–H bands stretching, respectively) was increased for eggs from challenged groups, especially for the Gram-negative group. Moreover, the bands at 2917 and 2850 cm^–1^ (C–H stretching), at 1575 cm^–1^ (N–H bending), and at 1539 cm^–1^ (asymmetric stretching carboxylate ion COO^–^ and C=C stretching) were more pronounced in the eggs from the challenged groups. Meanwhile, a decrease in the band intensity at 1370 cm^–1^ (C–H scissoring from alkanes) also occurred. The band at 1735 cm^–1^ (stretching of C=O from esters) showed greater intensity in the control group, while samples of eggs from the Gram-negative group showed lower intensity. The increased intensity of bands related to N–H bonds in eggs from challenged groups agrees with the high nitrogen content evidenced in EDS evaluations (Table 1).

The hexane egg extract (EE2) from all egg groups exhibited bands in the 2960–2860 cm^–1^ region (C–H stretching), at 1712 cm^–1^ (C=O stretching from carboxylic acids), at 1460 and 1370 cm^–1^ (C–H scissoring), and at 720 cm^–1^ (C–H rocking). The Gram-positive and Gram-negative groups also displayed bands at 3294 cm^–1^ (−OH stretching), 2145 cm^–1^ (−C≡C– stretching), 1640 cm^–1^ (N–H bending, C=O stretching from amides, and C=C stretching from alkenes), 1100 cm^–1^ (C–O stretching), 890 cm^–1^ (−C=C–H bending), and 660 cm^–1^ (−C–H bending from aromatic rings) (Figure 3B).

Otherwise, after the extraction process with hexane, similarities in the structural characteristics were observed among eggs from all groups (Figure 3C). At 3370 cm^–1^ (O–H) and 1735 cm^–1^ (C=O), the greatest intensities occurred for eggs from the control group. When the FTIR results of each group are compared (Figure 3D–F), the intensification of the bands related to the O–H (3370 cm^–1^), C=O (1735 cm^–1^), bending of the amide N–H (1640 cm^–1^), and carboxylate ion COO^–^ and C=C (1540 cm^–1^) is clear. Meanwhile, the intensities of the bands assigned to alkanes, C–C (2917 and 2850 cm^–1^) and C–H (1370 cm^–1^), were strongly decreased. All of these findings are in accordance with FEG-SEM images (Figure 1D–F,J–L,P,Q), proving the efficiency of mucus layer remotion.

### 2.3 Microbiological Activity of Extracts from *G. mellonella* Eggs

**Figure 4:**
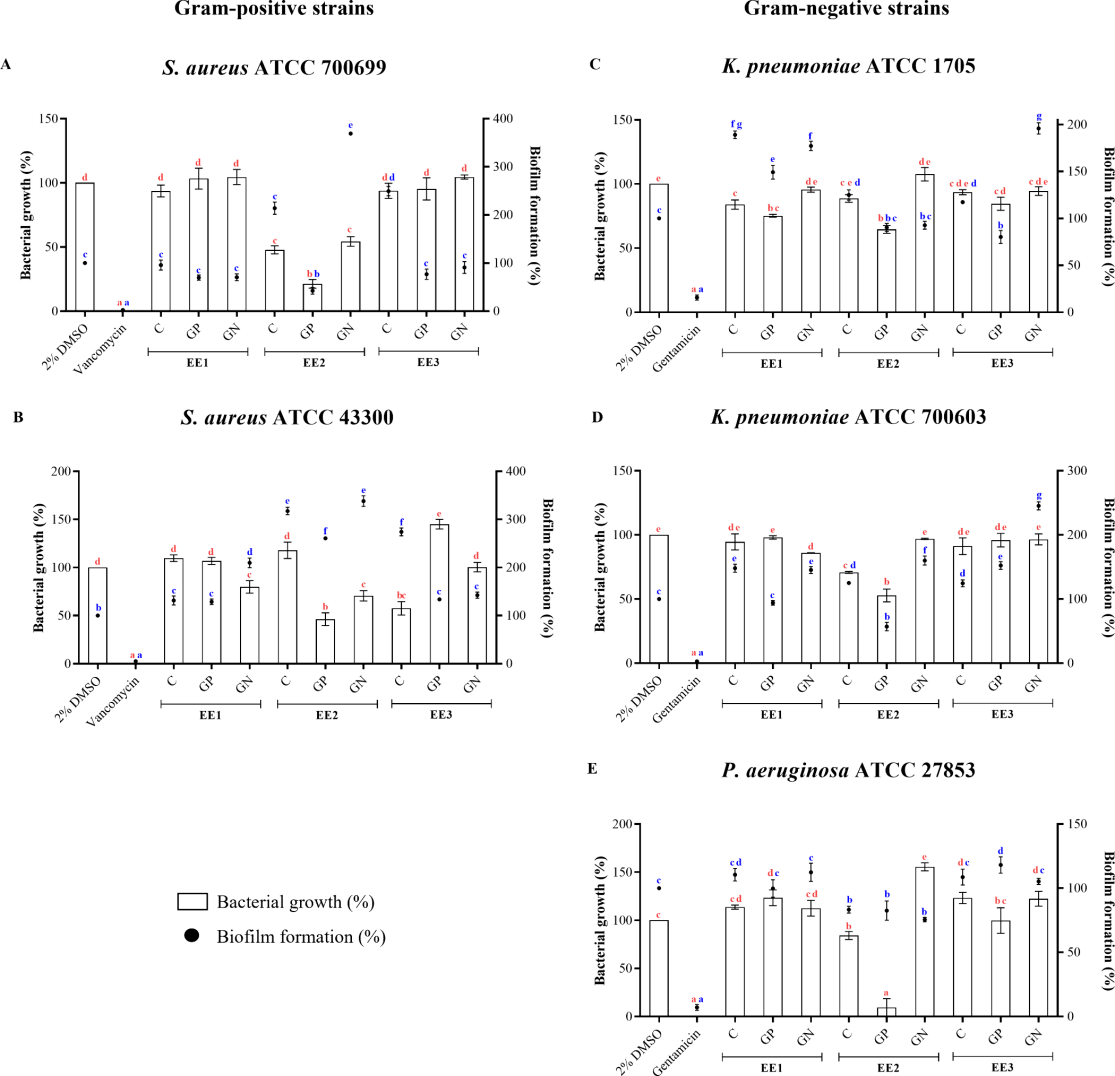
Biological activity of extracts from *G. mellonella* eggs at 350 μg/mL against Gram-positive (A and B) and Gram-negative (C–E) strains. *S. aureus* ATCC 700699 (A); *S. aureus* ATCC 43300 (B); **K. pneumoniae** ATCC 1705 (C); **K. pneumoniae** ATCC 7000603 (D); **P. aeruginosa** ATCC 27853 (E). Vehicle (2% DMSO) was used as negative control, while vancomycin and gentamicin were used as positive controls for antibacterial activity. Bars indicate bacterial growth (%) and circles indicate biofilm formation (%). Groups represented with the same letter indicate no significant difference using one-way ANOVA followed by Tukey’s post-test (*p*-value < 0.05). Letters in red and blue correspond to bacterial growth and biofilm formation, respectively.

The EE1 (total aqueous egg extract) and EE3 (aqueous egg extract without the mucous layer) extracts showed low activity against all strains (Figure 4). Otherwise, EE2 (hexane egg extract) extracts showed increased activity against all bacterial strains (Figure 4). These results indicated that the mucous layer, increased in eggs from larvae challenged with bacterial antigens (Figures 1 and 2), protects the offspring and can be a source of anti-infective compounds for multidrug-resistant bacteria.

Against the Gram-positive strains, EE2 extracts showed antibacterial activity (52, 46, and 79% growth inhibition for EE2C, EE2GN, and EE2GP, respectively) and only EE2GP presented antibiofilm activity (58% biofilm formation inhibition) against *S. aureus* ATCC 700699 (Figure 4A). For *S. aureus* ATCC 43300 (Figure 4B), EE2 extracts of eggs from challenged groups showed antibacterial action (29 and 54% growth inhibition for EE2GN and EE2GP, respectively). Regarding the three Gram-negative strains evaluated, **K. pneumoniae** ATCC 1705 had growth inhibited by 36% when treated with EE2GP (Figure 4C), **K. pneumoniae** ATCC 700603 had growth inhibited by EE2C and EE2GP (29 and 47%, respectively), and about 43% biofilm formation was prevented by EE2GP (Figure 4D). Furthermore, **P. aeruginosa** ATCC 27853 also had growth inhibited by 91% when exposed to EE2GP, and the biofilm formation was inhibited by all extracts (17, 24, and 17% for EE2C, EE2GN, and EE2GP, respectively) (Figure 4E).

Values exceeding 100% indicate stimulation of biofilm formation or bacterial growth compared to the negative control (2% DMSO). These effects may be attributed to the chemical nature of the extracts, which could (i) induce bacterial stress, leading to increased biofilm formation relative to the control,^6^ or (ii) provide microorganisms with substances that serve as a nutritional source, enhancing their growth beyond that of the negative control.

These results provide evidence that the response of *G. mellonella* to bacterial antigens, especially from Gram-positive strains, led to a significant broad-spectrum antibacterial activity against multidrug-resistant bacterial strains. In agreement with this work, *G. mellonella* larvae exposed to *S. aureus* antigens showed resistance to subsequent infections caused by both bacteria and fungi.^28^ Thus, the specific stimuli provided by *S. aureus* antigens protect *G. mellonella* from different types of microorganisms.

### 2.4 Identification of Volatile Substances from Extracts of *G. mellonella* Eggs

A higher yield was reached for EE2 extracts of eggs obtained from larvae exposed to Gram-negative bacterial antigens (EE2GN) when compared with the other groups (Table S1). It can be explained by the thickening of its mucous layer, as observed by FEG-SEM and histology analyses (Figures 1 and 2).

**Figure 5:**
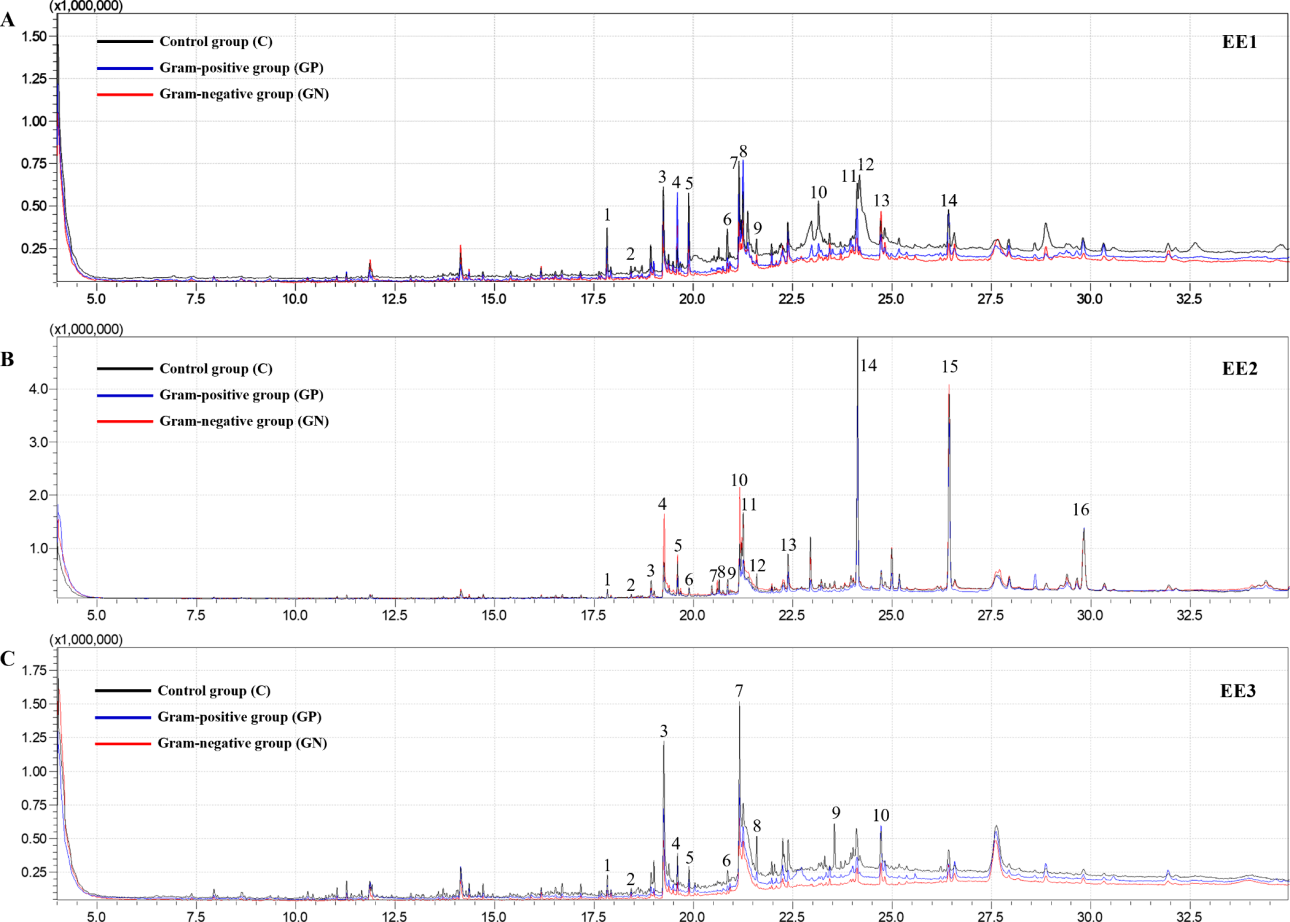
GC-MS chromatograms of *G. mellonella* egg extracts. Total aqueous extract from whole eggs (EE1) (A); hexane extract from the eggs (EE2) (B); serial aqueous extract from eggs without the mucous layer (EE3) (C). The control group is black, the Gram-positive group is blue, and the Gram-negative group is red.

Considering the nonpolar feature of EE2, the profile of volatile substances of all extracts (EE1, EE2, and EE3) was determined by GC-MS. 14, 16, and 10 substances were identified in EE1 (Figure 5A), EE2 (Figure 5B), and EE3 (Figure 5C), respectively (Table 2). The EE1 extract contains seven esters, three hydrocarbons, two aldehydes, one carboxylic acid, and one amide. Meanwhile, EE2 shows eight esters, four hydrocarbons, two aldehydes, and two carboxylic acids. These findings are reinforced by chemical bonds assigned for EE2 through FTIR analysis (Figure 3B). Interestingly, hydrocarbons (alkanes) are absent in the volatile profile of EE3, which agrees with the complete removal of the eggs’ mucous layer, as shown by FEG-SEM (Figure 1D–R) and FTIR (Figure 3D–F). Otherwise, these samples showed five esters, two aldehydes, two carboxylic acids, and one phenol derivate.

**Table 2:** Volatile Substances Profile of *G. mellonella* Egg Extracts (EE) by GC-MS^a^

			**peak area (%)**					
	**peak number**	**RT (min)**	**C**	**GP**	**GN**	**compound name**	**MW**	**formula**
EE1	1	17.835	3.8	4.8	5.1	tetradecanal	212	C_14_H_28_O
	2	18.935	2.8	1.9	2.8	tridecanoic acid, methyl ester	228	C_14_H_28_O_2_
	3	19.250	9.7	13.0	14.7	hexadecanoic acid, methyl ester	270	C_17_H_34_O_2_
	4	19.600	1.8	10.4	9.1	hexadecanoic acid, ethyl ester	284	C_18_H_36_O_2_
	5	19.880	6.8	7.3	7.6	octadecanal	268	C_18_H_36_O
	6	20.855	2.4	1.7	1.8	methyl stearate	298	C_19_H_38_O_2_
	7	21.155	9.3	12.0	11.8	octadecanoic acid	284	C_18_H_36_O_2_
	8	21.250.	7.4	19.2	13.8	ethyl oleate	310	C_20_H_38_O_2_
	9	21.595	1.5	1.5	3.8	heptacosyl acetate	438	C_29_H_58_O_2_
	10	23.150	12.0	3.4	2.4	nonadecanamide	297	C_19_H_39_NO
	11	24.105	8.7	9.4	7.6	heneicosane	269	C_21_H_44_
	12	24.185	25.6	1.5	2.5	dotriacontane	450	C_32_H_66_
	13	24.725	3.1	4.5	11.9	bis(2-ethylhexyl) phthalate	390	C_24_H_38_O_4_
	14	26.405	5.0	9.5	5.1	tetracontane	562	C_40_H_82_
EE2	1	17.840	0.7	0.9	0.6	tetradecanal	212	C_14_H_28_O
	2	18.435	0.3	0.3	0.2	1,2-benzenedicarboxylic acid, bis(2-methylpropyl) ester	278	C_16_H_22_O_4_
	3	18.935	1.5	0.9	0.7	hexadecanoic acid, methyl ester	270	C_17_H_34_O_2_
	4	19.255	4.0	5.0	8.9	*n*-hexadecanoic acid	256	C_16_H_32_O_2_
	5	19.600	2.9	2.2	3.4	hexadecanoic acid, ethyl ester	284	C_18_H_36_O_2_
	6	19.895	0.7	1.0	0.8	octadecanal	268	C_18_H_36_O
	7	20.600	0.6	0.5	1.1	9,12-octadecadienoic acid (*Z*,*Z*)–, methyl ester	294	C_19_H_34_O_2_
	8	20.645	1.6	0.8	1.2	9-octadecenoic acid, methyl ester, (*E*)–	296	C_19_H_36_O_2_
	9	20.860	1.5	0.9	0.7	methyl stearate	298	C_19_H_38_O_2_
	10	21.165	5.3	5.6	9.6	octadecanoic acid	284	C_18_H_36_O_2_
	11	21.255	10.5	6.2	9.3	ethyl oleate	310	C_20_H_38_O_2_
	12	21.595	2.2	1.2	0.3	heptacosyl acetate	438	C_29_H_58_O_2_
	13	22.360	4.7	3.3	3.7	heneicosane	296	C_21_H_44_
	14	24.105	25.7	25.7	21.3	pentacosane	352	C_25_H_52_
	15	26.405	25.9	29.8	25.8	dotriacontane	450	C_32_H_66_
	16	29.770	11.9	15.8	12.4	tetracontane	562	C_40_H_82_
EE3	1	17.835	2.5	3.0	2.8	tetradecanal	212	C_14_H_28_O
	2	18.435	1.0	1.2	1.5	1,2-benzenedicarboxylic acid, bis(2-methylpropyl) ester	278	C_16_H_22_O_4_
	3	19.255	28.8	25.7	25.9	*n*-hexadecanoic acid	256	C_16_H_32_O_2_
	4	19.600	5.1	7.3	5.9	hexadecanoic acid, ethyl ester	284	C_18_H_36_O_2_
	5	19.890	2.8	2.8	3.6	hexadecanal	240	C_16_H_32_O
	6	20.895	1.9	1.0	1.1	methyl stearate	298	C_19_H_38_O_2_
	7	21.150	38.1	40.6	46.0	octadecanoic acid	284	C_18_H_36_O_2_
	8	21.590	5.6	3.5	3.6	heptacosyl acetate	438	C_29_H_58_O_2_
	9	23.550	6.7	-	-	phenol, 2,2′-methylenebis[6-(1,1-dimethylethyl)-4-methyl	340	C_23_H_32_O_2_
	10	24.720	7.5	15.0	9.6	bis(2-ethylhexyl) phthalate	390	C_24_H_38_O_4_

a RT: retention time; MW: molecular weight; -: absence; C: eggs of larvae from the control group; GP: eggs of larvae that received antigens of Gram-positive bacteria; and GN: eggs of larvae that received antigens of Gram-negative bacteria.

Hydrocarbons (alkanes) are the major compounds in EE2 extracts, comprising 63.2 to 74.6% of samples. Among them, dotriacontane and tetracontane were increased in the Gram-positive group’s eggs compared to eggs from the control and Gram-negative groups. Otherwise, long-chain fatty acids, such as *n*-hexadecanoic acid (palmitic acid) and octadecanoic acid (stearic acid), were the major compounds in the EE3 extracts, ranging from 66.3 to 71.9%. The EE1 extracts showed a greater diversity of substances, including those present in EE2 and EE3 (Table 2).

Several fatty acids, including *n*-hexadecanoic and octadecanoic acids, and long-chain hydrocarbons, including dotriacontane and tetracontane, are recognized by their antimicrobial properties, typically presenting a broad spectrum of action. However, their activities are reported from bioassay-guided fractionation of extracts obtained from a variety of organisms rather than from pure compounds.^29−33^ The *n*-hexadecanoic acid represents an exception. It showed moderate antibacterial activity at 50 μg/mL against the Gram-positives *S. aureus* and **B. subtilis**, and the Gram-negatives **E. coli** and **K. pneumoniae*.*(34) Furthermore, when films of *n*-hexadecanoic and octadecanoic acids were recrystallized on the surface of highly ordered pyrolytic graphite, the material exhibited bactericidal activity against **P. aeruginosa** and *S. aureus*.^35^ These findings shed light on the investigation of long-chain hydrocarbons as single antibacterial agents.

## 3 Conclusions

This study is the first to report the anti-infective activity of *Galleria mellonella* eggs against clinically relevant bacteria, including multidrug-resistant *Staphylococcus aureus*, *Klebsiella pneumoniae*, and *Pseudomonas aeruginosa*. Notably, when larvae were exposed to bacterial antigens, a stimulus in the antibacterial activity of the eggs was observed, particularly in the mucous layer, suggesting enhanced protection. Although the most significant increase in mucous layer thickness was observed in eggs from larvae challenged with Gram-negative antigens, broad-spectrum antibacterial activity was verified in the mucous layer of the Gram-positive group. This activity can be attributed to the chemical composition of the mucous layer extracts, which are rich in hydrocarbons (comprising 63–75% of the composition). In particular, the mucous layer from the Gram-positive group contained high levels of dotriacontane and tetracontane, which together accounted for nearly half of the chemical composition.

This study advances the understanding of *G. mellonella* biology and represents a pioneering effort in the physicochemical characterization of this insect’s eggs, including the alterations induced by larval exposure to antigens from multidrug-resistant bacteria. Furthermore, the findings highlight *G. mellonella* eggs as a source of bioactive compounds and underscore the potential of long-chain hydrocarbons, such as dotriacontane and tetracontane, for the development of novel antibacterial agents.

## 4 Methods

### 4.1 *Galleria mellonella*

The lifecycle of *Galleria mellonella* was maintained under controlled laboratory conditions. Moths, eggs, and larvae were kept at temperatures of 22, 28, and 32 °C, respectively. The larvae were fed a laboratory diet consisting of wheat flour (14.7%), coarse wheat bran (14.7%), wheat germ (14.7%), powdered milk (29.4%), liquid honey (8.8%), glycerol (8.8%), and brown sugar (8.8%).

### 4.2 Bacterial Strains

This study utilized the following bacterial strains:

(i) Gram-positive strains: *Staphylococcus aureus* ATCC 43300 (methicillin-resistant *Staphylococcus aureus*, MRSA) and ATCC 700699 (MRSA and vancomycin-intermediate *Staphylococcus aureus*, VISA); and
(ii) Gram-negative strains: *Klebsiella pneumoniae*ATCC BAA-1705 (carbapenem-hydrolyzing β-lactamase gene, *bla*KPC positive; New Delhi metallo-β-lactamase gene, *bla*NDM negative), ATCC 700603 (extended-spectrum β-lactamases, ESBL), and *Pseudomonas aeruginosa* ATCC 27853.

### 4.3 Challenge of *G. mellonella* with Bacterial Antigens

To potentially stimulate the production of anti-infective compounds, groups of larvae were challenged with suspensions of Gram-positive or Gram-negative bacterial antigens via systemic inoculation. All bacterial strains were cultured from stock on Mueller-Hinton (MH) agar, except for *S. aureus* ATCC 700699, which was cultured on brain heart infusion (BHI) agar supplemented with 4 μg/mL vancomycin.

Bacterial suspensions were prepared in sterile 0.9% NaCl solution to an optical density (OD) of 0.150 at 620 nm and measured using a SpectraMax M2e spectrophotometer (Molecular Devices Corporation, USA), corresponding to approximately 10^8^ colony-forming units (CFU)/mL. Serial dilutions of the initial suspension were prepared and plated on MH agar. After 24 h of incubation at 37 °C, colony-forming units (CFU) were counted to determine the suspension concentrations. Gram-positive antigen suspension was obtained by mixing equal volumes (1:1) of each Gram-positive bacterial inoculum and autoclaved (121 °C, 1.1 atm, 15 min) to produce a cell lysate. The same procedure was followed for the Gram-negative antigen suspension, mixing equal volumes (1:1:1) of each Gram-negative bacterial inoculum.

Groups of 200 larvae, each weighing 200–250 mg (6th instar), were challenged through two inoculations of 10 μL of bacterial antigen suspension using a Hamilton syringe (Sigma-Aldrich, Germany) in the last proleg, with a 48 h interval between applications. Three experimental groups were established:

(i) Larvae receiving Gram-positive bacterial antigens (GP);
(ii) larvae receiving Gram-negative bacterial antigens (GN); and
(iii) Control group receiving 0.9% NaCl solution (C).

All groups were monitored until the completion of their lifecycle. Eggs obtained from intragroup mating were collected and stored at −20 °C until use.

### 4.4 Morphological and Physicochemical Characterization of *G. mellonella* Eggs

#### 4.4.1 Histology

Egg samples underwent dehydration, diaphanization, and paraffin embedding, followed by microtomy (5 μm sections). The sections were stained with Hematoxylin and Eosin (HE), and slide images were captured using the EVOS FL Auto 2 microscope (Thermo Fisher Scientific, USA). Mucous layer thickness was measured at six distinct points on each sample using ImageJ software (National Institutes of Health, USA).

#### 4.4.2 Field Emission Gun Scanning Electron Microscopy (FEG-SEM) Coupled with Energy-Dispersive X-ray Spectroscopy (EDS)

FEG-SEM coupled with EDS was employed to visualize morphological changes on the eggs’ surface before and after exposure to bacterial antigens. Eggs were fixed in 2.5% glutaraldehyde in 0.1 M phosphate buffer (pH 7.4), washed in phosphate buffer, and treated with 1% osmium tetroxide in phosphate buffer. Samples were dehydrated in an acetone gradient and subjected to critical point drying.

Dehydrated samples were mounted onto metal supports (stubs) with double-sided carbon tape and coated with a thin layer of gold. FEG-SEM analysis was performed on a FEI Inspect F50 microscope (FEI Company, USA) in secondary electron mode at 20 kV and a spot size of 3.0. EDS analysis was conducted to determine the surface elemental composition of the samples.

#### 4.4.3 Fourier Transform Infrared Spectroscopy (FTIR)

FTIR was used to analyze chemical composition changes in *G. mellonella* eggs before and after exposure to bacterial antigens. Spectra were obtained using the Spectrum One FT-IR spectrometer (PerkinElmer Instruments, USA) equipped with a universal attenuated total reflectance (UATR) accessory. Data were acquired in the wavenumber range of 4000 to 650 cm^–1^ with 12 scans per sample.

### 4.5 Chemical Extraction of *G. mellonella* Eggs

Eggs were subjected to various extraction processes using methodologies adapted from the literature.^15,36,37^ In a 50 mL conical tube, 1000 mg of eggs and 1.5 mL (repeated twice) of 10 mM sodium phosphate buffer (pH 7.3) were combined. The mixture was centrifuged at 5000 rpm at 18 °C for 20 min. The aqueous supernatant was transferred to a new tube, frozen at −20 °C, and subsequently lyophilized to obtain the total aqueous egg extract (EE1).

**Figure 6:**
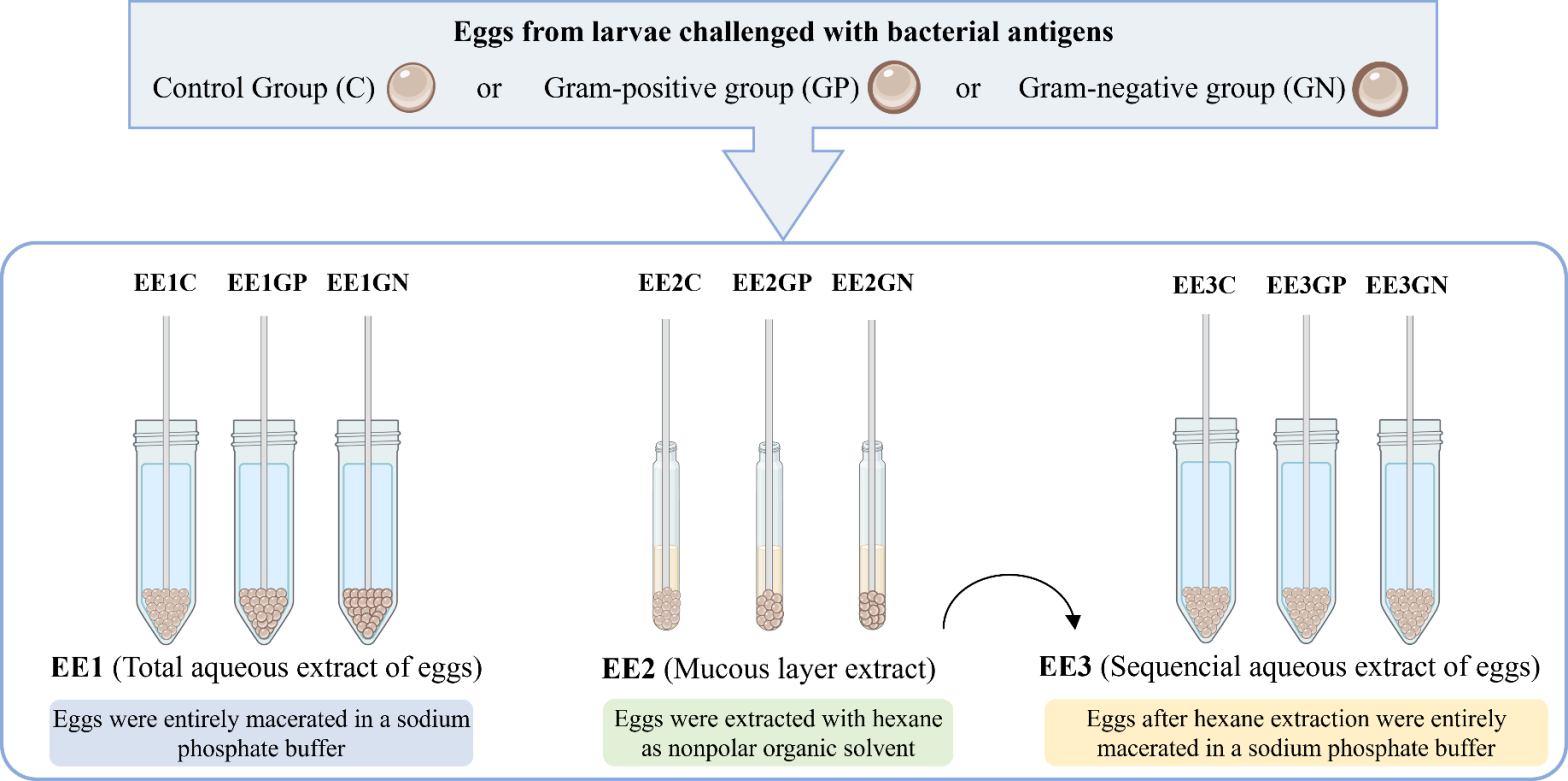
Schematic illustration of extracts prepared from *G. mellonella* eggs. Eggs obtained from larvae that received injection of saline (control group, C) or antigens from Gram-positive (GP) or Gram-negative (GN) bacteria were submitted to aqueous extraction (EE1) or organic extraction (EE2) followed by sequential aqueous extraction (EE3).

A serial extraction was performed on another batch of eggs to assess the role of the mucous layer in the biological activity. Eggs (1000 mg) were immersed in 5 mL (repeated twice) of hexane^13^ and gently agitated on a rotating platform at 60 rpm for 30 min. The supernatant was transferred to a glass tube, and the solvent was removed by evaporation to obtain hexane egg extract (EE2). Residual solvent was removed from the eggs by washing with 5 mL of ultrapure water, which was subsequently discarded. The eggs, now devoid of the mucous layer, were macerated in 10 mM sodium phosphate buffer (pH 7.3) and extracted as described for EE1 to obtain the aqueous egg extract without a mucous layer (EE3). The material was frozen at −20 °C and lyophilized. Extracts from the control group and those challenged with bacterial antigens were named as described in Figure 6. Chemical profile of EE2 extracts was evaluated by FTIR as described in Section 4.4.3.

Changes in the surface morphology and structural characteristics of *G. mellonella* eggs before and after chemical extraction were analyzed using FEG-SEM and FTIR, respectively, as outlined in Sections 4.4.2 and 4.4.3.

#### 4.5.1 Identification of Volatile Substances from *G. mellonella* Eggs

All dried extracts were redissolved in hexane at a concentration of 0.5 mg/mL and analyzed using gas chromatography–mass spectrometry (GC-MS) to identify volatile nonpolar substances. The analysis was performed on a GC-MS-QP-2010-Plus system coupled with a mass spectrometer (Shimadzu, Japan) using an OV-5 capillary column (5% diphenyl, 95% dimethylpolysiloxane stationary phase) measuring 30 m × 0.25 mm × 0.25 μm (Ohio Valley Specialty Company, USA). Ultrapure helium served as the carrier gas at a flow rate of 1.03 mL/min. Injections were carried out at 270 °C in splitless mode.

The oven-heating program consisted of a 10 °C/min ramp from 40 to 270 °C, followed by a 12 min hold at 270 °C. The interface and ionization source were maintained at 270 °C throughout the analysis. The mass spectrometer operated in SCAN acquisition mode, covering *m*/*z* ratios from 40 to 400 and utilizing electron impact ionization at 70 eV. Peaks with a similarity index above 90% were identified by using the NIST11s.lib library. The percentage composition of the identified substances was calculated by comparing the peak area of each substance to the total peak area of all identified substances. All analyses were performed by using GCMSsolution software (Shimadzu, Japan).

### 4.6 Anti-Infective Activities

#### 4.6.1 Bacterial Culture Conditions and Sample Preparation

The Gram-positive and Gram-negative strains were grown from the skim milk stock on MH agar, with the exception of *S. aureus* ATCC 700699, which was grown on BHI agar supplemented with 4 μg/mL vancomycin. Bacterial suspensions were prepared independently in sterile 0.9% NaCl solution until reaching an OD at 620 nm of 0.150, which corresponds to approximately 1 × 10^8^ CFU/mL. Bacterial growth and biofilm formation for each strain were evaluated using BHI broth (for both *K. pneumoniae*strains), BHI broth supplemented with 2% glucose (for both *S. aureus* strains), and Tryptic Soy Broth (TSB; for *P. aeruginosa* strain).

Egg extracts were dissolved in dimethyl sulfoxide (DMSO; 99.5%, Sigma-Aldrich, USA) and tested at a final concentration of 350 μg/mL.

#### 4.6.2 Antibacterial Activity

Antibacterial activity was assessed using the broth microdilution method in sterile 96-well plates (Costar Corning 3599, USA). Each well contained 80 μL of bacterial suspension, 80 μL of egg extract, and 40 μL of the culture medium. Plates were incubated at 37 °C for 24 h. Bacterial growth was determined by measuring the OD at 620 nm at the start (0 h) and end (24 h) of the incubation.

Negative controls consisted of 2% DMSO (vehicle), while positive controls included 16 μg/mL vancomycin for *S. aureus* ATCC 700699, 8 μg/mL vancomycin for *S. aureus* ATCC 43300, 8 μg/mL gentamicin for *P. aeruginosa*, and 16 μg/mL gentamicin for both *K. pneumoniae*strains (Sigma-Aldrich, USA). Negative controls were considered as representing 100% bacterial growth.^34,35^ Each assay was performed at least in triplicate.

#### 4.6.3 Antibiofilm Formation Activity

Antibiofilm activity was evaluated by using the crystal violet staining technique in sterile 96-well plates. Following incubation as described in Section 4.6.2, the well contents were removed, and wells were gently washed three times with 200 μL of sterile 0.9% NaCl solution to remove nonadherent planktonic cells. Biofilms were fixed at 60 °C for 60 min and stained with 200 μL of 0.4% crystal violet for 15 min at room temperature. Wells were washed with water, and 200 μL of 99.5% ethanol was added to solubilize the dye for 30 min. Absorbance was measured at 570 nm. Negative controls were considered as 100% biofilm formation.^35^ Each assay was performed at least in triplicate.

### 4.7 Statistical Analysis

Histological measurements and anti-infective assay results were analyzed using one-way ANOVA followed by Tukey’s post-test in GraphPad Prism 8.0.2 (USA). A *p*-value ≤ 0.05 was considered statistically significant.

## Supporting Information Available

(i) Methods; (ii) Figure S1, which shows FEG-SEM images of the *G. mellonella* eggs surface after exposition to *S. aureus*; (iii) Figure S2, which presents the EDS spectra of the outer surface of *G. mellonella* eggs; and (iv) Table S1, which provides the yield of *G. mellonella* egg extractions (PDF)
