# Correction to “Long-Chain Hydrocarbons in the Mucous Layer of the *Galleria mellonella* Insect Eggs as Potential Antibacterial Agents against Multidrug-Resistant Bacteria”

**DOI:** 10.1021/acsomega.5c07768

**Published:** 2025-08-12

**Authors:** Letícia F. Luz, Gabriela L. Nascimento, Gabrielle N. Volcan, Rosane A. Ligabue, Gabriela M. Miranda, Danielle S. Trentin

**Funding**

This work was supported by the Federal University of Health Sciences of Porto Alegre (Programa de Fomento para a Realização de Projetos de Pesquisa na UFCSPA - Edital PROPPG no. 24/2023) and the Conselho Nacional de Desenvolvimento Científico e Tecnológico (CNPq), Brazil (grant number 308617/2021-5). This study is part of the National Institute of Science and Technology in 3D printing and Advanced Materials Applied to Human and Veterinary Health - INCT _3D-Saúde, funded by CNPq, Brazil (grant number 406436/2022-3). L.F.L. and G.M.M. acknowledge the Coordenação de Aperfeiçoamento de Pessoal de Nivel Superior (CAPES), Brazil, and CNPq for their fellowships. The article processing charge for the publication of this research was funded by the CAPES, Brazil (ROR identifier: 00x0ma614).

