# Supplementary material for: Long-Chain Hydrocarbons in the Mucous Layer of the Galleria mellonella Insect Eggs as Potential Antibacterial Agents against Multidrug-Resistant Bacteria
Source: ACS Omega. 2025 Apr 28;10(18):18899–909. doi: 10.1021/acsomega.5c00938 (PMC12079585; doi:10.1021/acsomega.5c00938)
Supplement: Supplementary file 1 — ao5c00938_si_003.pdf [file ao5c00938_si_003.pdf]

Supporting information

Long-chain hydrocarbons in the mucous layer of the *Galleria mellonella* insect eggs as potential antibacterial agents against multidrug-resistant bacteria

Letícia F. Luz[1], Gabriela L. Nascimento[2], Gabrielle N. Volcan[3], Rosane A. Ligabue[4], Gabriela M. Miranda[1], Danielle S. Trentin[1]

[1] Federal University of Health Sciences of Porto Alegre, Graduate Program in Biosciences, Sarmento Leite, 245, 90050-170, Porto Alegre, Rio Grande do Sul, Brazil.

[2] Federal University of Health Sciences of Porto Alegre, Undergraduate Nursing Course, Sarmento Leite, 245, 90050-170, Porto Alegre, Rio Grande do Sul, Brazil.

[3] Federal University of Health Sciences of Porto Alegre, Undergraduate Pharmacy Course, Sarmento Leite, 245, 90050-170, Porto Alegre, Rio Grande do Sul, Brazil.

[4] Pontifical Catholic University of Rio Grande do Sul, Graduate Program in Materials Engineering and Technology, Ipiranga, 6681, 90619-900, Porto Alegre, Rio Grande do Sul, Brazil.

Methods

1. Preliminary evaluation of *G. mellonella* eggs exposition to *S. aureus*:

List of Figures

Figure S1. FEG-SEM images of the *G. mellonella* eggs surface after exposition to *S. aureus*.

Figure S2. EDS spectra of the outer surface of *G. mellonella* eggs (A-C)

List of Tables

Table S1. Yields from *G. mellonella* egg extractions

Methods

1. Preliminary evaluation of G. mellonella eggs exposition to S. aureus

Bacterial suspension of *S. aureus* ATCC43300 was prepared in sterile 0.9% NaCl solution to an optical density (OD) of 0.150 at 620 nm, measured using a SpectraMax M2e spectrophotometer. Plates of Mueller-Hinton agar were inoculated with bacterial suspension using a sterile swab. Then, about 8 mg of each group of eggs (mimicking antimicrobial susceptibility disks = 5 mm diameter) were added in triplicate. The plates were incubated at 37°C for 24 h. Afterwards, the eggs were recovered and fixed in 2.5% glutaraldehyde in 0.1 M phosphate buffer (pH 7.4). Biological preparation and FEG-SEM analysis were performed as described in the section 4.4.3 of manuscript.

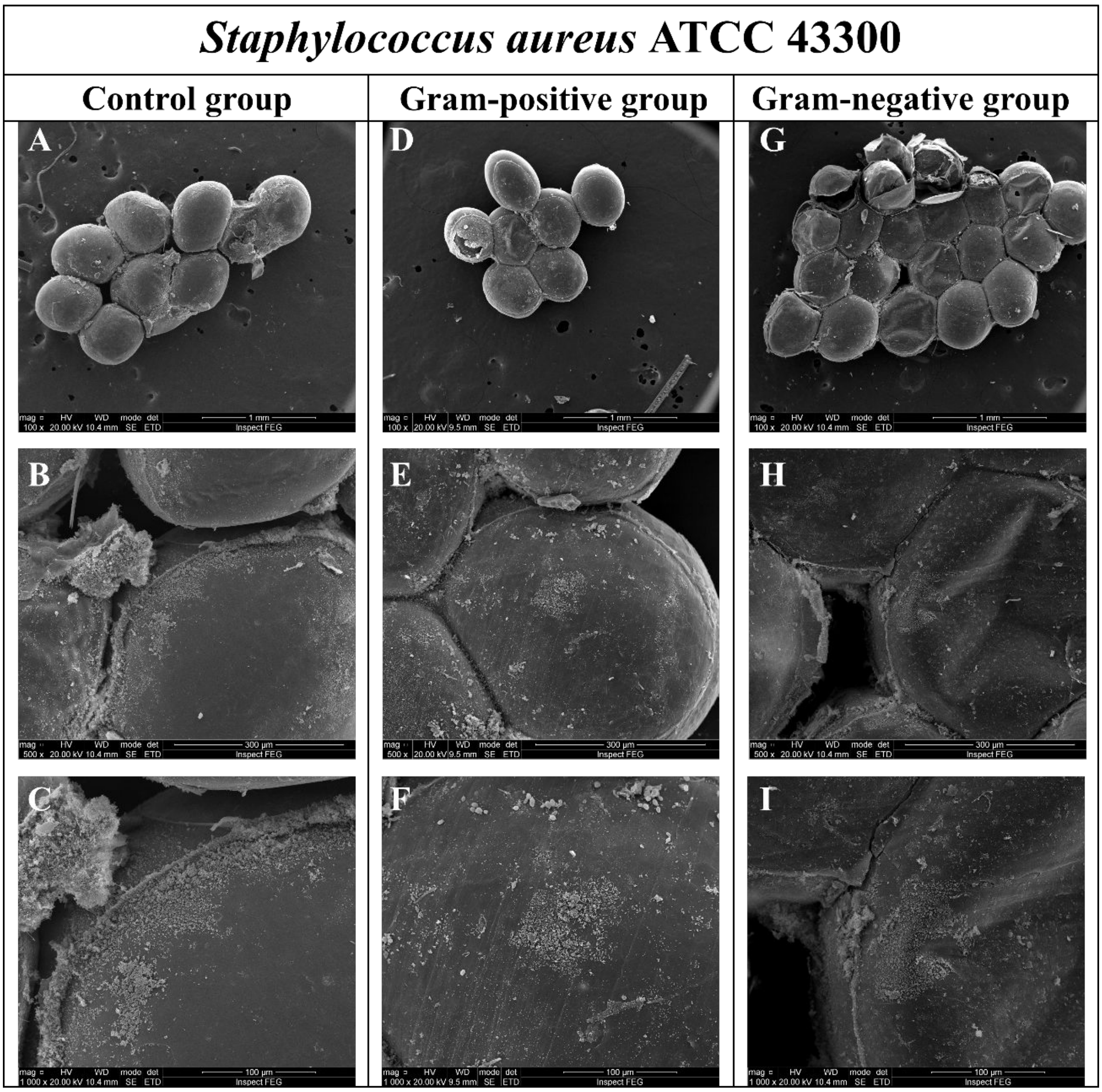

Figure S1. FEG-SEM images of the *G. mellonella* eggs surface after exposition to *S. aureus*: Eggs from the control group (A-C); eggs from larvae exposed to Gram-positive antigens (D-F); and eggs from larvae exposed to Gram-negative antigens (G-I). Scale bars in images – A, D, G: 1 mm; B, E, H: 300 µm; C, F, I: 100 µm.

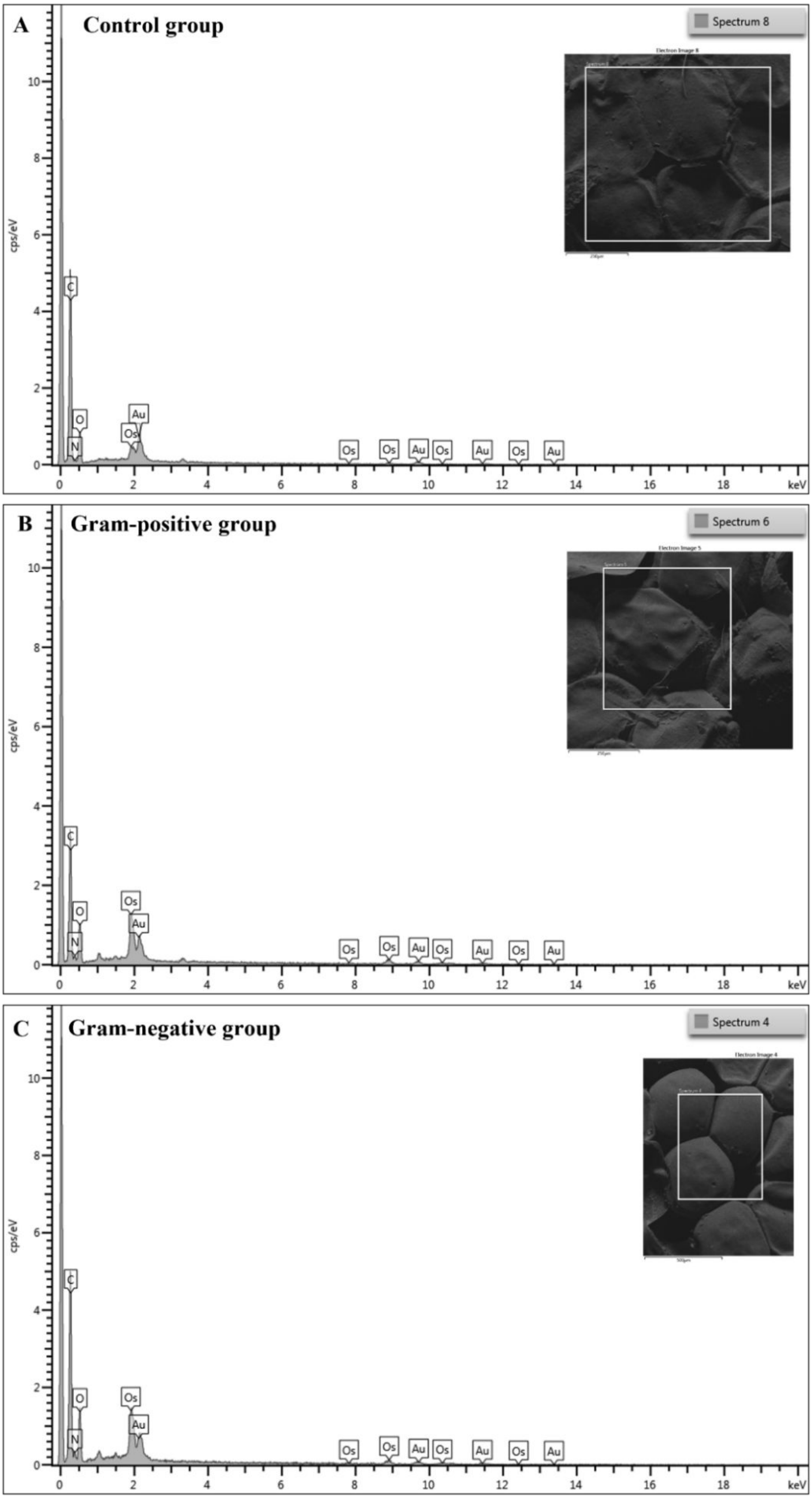

Figure S2. EDS spectra showing the surface elementary composition of *G. mellonella* eggs (A-C). Control group (A); Gram-positive group (B); Gram-negative group (C). Osmium (Os) and Gold (Au) are present due to the use of osmium tetroxide and gold during sample preparation.

Table S1. Percentual yield of *G. mellonella* egg extractions.

Sample	Initial mass (mg)	Final mass (mg)	Yield (%)
**EE1C**	1000.0	25.1	2.5
**EE1GP**	1000.0	28.9	2.9
**EE1GN**	1000.0	19.1	1.9
**EE2C**	1000.0	4.5	0.5
**EE2GP**	1000.0	5.1	0.5
**EE2GN**	1000.0	20.5	2.1
**EE3C**	995.5	24.2	2.4
**EE3GP**	994.9	37.7	3.8
**EE3GN**	979.5	42.1	4.3

C: eggs of larvae from the control group; GP: eggs of larvae that received antigens of Gram-positive bacteria; and GN: eggs of larvae that received antigens of Gram-negative bacteria.
